# CIS8/469: Introduction of New Information and Communication Technology into the Workflow of General Practitioners - Examination of the consequences on the interaction between doctor and patient

**DOI:** 10.2196/jmir.1.suppl1.e13

**Published:** 1999-09-19

**Authors:** A Vocke, HB Bludau, N Schaper, W Herzog

**Affiliations:** ^1^Universität Heidelberg, HeidelbergGermany

**Keywords:** Integrated Advanced Information Management Systems, Physicians, Doctor-Patient-Interaction, IuK-Technologies

## Abstract

**Introduction:**

Since 1997 an interdisciplinary research project is taking place at the Heidelberg University clinics with the subject "Mobile communication tools at the clinical practice". It was focused mainly on the introduction of new technologies to support stationary as well as ambulant medical and nursing occupation. Within the frame of construction of new information systems at hospitals the research works concentrated on aspects as the access to data banks of medical knowledge, electronic file management, picture transmission and knowledge based decision support. In the field of interpersonal communication the use of mobile radio equipment for the duty in service was to the fore. One of the main questions concerns the forecast of psychosocial technical consequences.

**Methods:**

The research on hand focuses on consequences of new luK-technologies on the interaction between doctor and patient on the field of the general practitioner. An IST-SOLL analyses was made among general practitioners, internists, paediatricians, dentists (N=48) and their patients (N=116) by means of half-structured interviews and questionnaires. With the instruments it was at first possible to make out a judgement of doctors and patients in view of their doctor-patient interaction and the actual technical status quo. In addition they inquired an assessment of the person what changes they would expect on the corresponding fields by the introduction of new technologies.

**Results:**

The results show that negative influences of new technologies on the doctor-patient interaction are expected by 18.5% of the questioned doctors and only 4.4% of the questioned patients. Doctors would approve the introduction of new technologies on condition that it would be user-friendly and that data protection, restriction to main functions as well as data exchange with clinics and other medical institutions would be guaranteed.

**Discussion:**

The results of the study are to be regarded as prognostics with regard to the development and introduction of new technologies. Statements will be possible in two areas:

1. Consequences on the contact between doctor and patient
2. Which innovations are regarded as useful by the doctors and which aspects should be taken into consideration in case of construction?

